# Improvement and prediction of secondary metabolites production under yeast extract elicitation of *Azadirachta indica* cell suspension culture using response surface methodology

**DOI:** 10.1186/s13568-021-01203-x

**Published:** 2021-03-17

**Authors:** Reza Farjaminezhad, Ghasemali Garoosi

**Affiliations:** Department of Biotechnology, Faculty of Agriculture and Natural Resources, Imam Khomeini International University (IKIU), P. O. Box 288, 34149-16818 Qazvin, Islamic Republic of Iran

**Keywords:** Azadiractin, Callus induction, Medicinal plant, Mevalonic acid, Neem, Squalene

## Abstract

Neem is a medicinal plant used as antimalarial, antibacterial, antiviral, insecticide, and antimicrobial drug. This study aimed to investigate and predict the effect of yeast extract and sampling time on cell growth, secondary metabolites synthesis, *SQS1* and *MOF1* genes expression by response surface methodology. The highest fresh and dry cell weights were 580.25 g/L and 21.01 g/L, respectively obtained 6 days after using 100 mg/L yeast extract. The highest azadirachtin accumulation and production were 16.08 mg/g DW and 219.78 mg/L obtained 2 and 4 days, respectively after using 25 mg/L yeast extract. Maximum mevalonic acid accumulation (1.75 mg/g DW) and production (23.77 mg/L) were observed 2 days after application of 50 mg/L yeast extract. The highest amount of squalene accumulation (0.22 mg/g DW) and production (4.53 mg/L) were achieved 4 days after using 50 mg/L yeast extract. Prediction results exhibited the highest azadirachtin accumulation (13.61 mg/g DW) and production (190.50 mg/L), mevalonic acid accumulation (0.50 mg/g DW) and production (5.57 mg/L), and squalene accumulation (0.30 mg/g DW) by using 245 mg/L yeast extract for 2 days, 71 mg/L yeast extract for 2 days, 200 mg/L yeast extract for 4.96 days, without yeast extract for 6.54 days and 4 days, respectively. Also, it was predicted that the highest squalene production is achieved by long-term exposure to high concentrations of yeast extract. The qRT-PCR analysis displayed the maximum relative gene expression of *SQS1* and *MOF1* by using 150 and 25 mg/L yeast extract for 4 and 2 days treatment.

**Supplementary Information:**

The online version contains supplementary material available at 10.1186/s13568-021-01203-x.

## Introduction

Plants are a rich source of medicinal compounds which are used to make medicine. About a quarter of the drugs approved by the Food and Drug Administration and European Medicines Agency are produced from plants, which shows their importance (Borkotoky and Banerjee 2020; Thomford et al. 2018). Neem (*Azadirachta indica*) is a member of the Meliaceae family and very important in traditional medicine. Studies have shown that neem leaf extract has free radical scavenging and anti-inflammatory activity and inhibits HSV-1 and MHV viruses (Alzohairy 2016; Sarkar et al. 2020; Tiwari et al. 2010). It has been reported that neem leaf extract can increase immunity against HIV/AIDS by increasing CD4^+^ cell levels (Mbah et al. 2007). Also, it has been reported that neem extract may be used against the COVID-19 infection (Roy and Bhattacharyya 2020). Neem tree has a variety of secondary metabolites, including azadirachtin, mevalonic acid, squalene, nimbin, nimbiol, polyphenolic flavonoids, etc. (Borkotoky and Banerjee 2020; Farjaminezhad and Garoosi 2020).

Secondary metabolites are not necessary to maintain the plant life cycle but play an important role in environmental adaptation (Park et al. 2020). Secondary metabolites have various uses and are mainly used as drugs, flavorings, fragrances, pigments, bio-pesticides, and food additives (Murthy et al. 2014). Studies show that the production of secondary metabolites depends on plant genetics, environmental factors, climate, season, growth period, plant parts, pre- and post-harvest processes, and extraction methods (Açıkgöz 2020). All of these factors create problems in the production of secondary metabolites in the traditional method and increases the cost of production (Ramachandra Rao and Ravishankar 2002). Therefore, plant tissue culture is used to produce secondary metabolites due to its reliability and predictability, independence from geographical, seasonal and environmental factors, modification or elimination of unwanted taste, and production of high quality and standard product (Abd El-Salam et al. 2015). Cell suspension culture is the best option to increase the production of secondary metabolites and respond to the increasing industrial demand for secondary metabolites (Rani et al. 2020).

There are several strategies to improve the production of secondary metabolites in plant tissue and cell culture (Park et al. 2020). One of these strategies is the use of elicitors. Elicitors are compounds that stimulate the production of secondary metabolites by biochemical changes in the plant (Namdeo 2007). Elicitors do this by activating the signal transduction cascades (Karalija et al. 2020). These signals act as stress factors and regulate enzyme activity and the production of secondary metabolites (Sharma and Agrawal 2018). Elicitors are divided into two types depending on the origin including biotic and abiotic. Biotic elicitors are produced from microbial or plant sources. Yeast extract is one of the biotic elicitors which derived from microbial sources (Ramirez-Estrada et al. 2016). Yeast extract contains various compounds such as chitin, β-glucan, glycopeptides, and ergosterol that are involved in plant defense responses (Baenas et al. 2014). It is one of the most common natural elicitors used in in vitro culture to induce secondary metabolites production (Cheng et al. 2013; Karalija et al. 2020). It has been used also successfully in various studies to improve the production of secondary metabolites such as rosmarinic acid (Gonçalves et al. 2019), sanguinarine (Guízar-González et al. 2016), xanthone (Krstić-Milošević et al. 2017) isoflavonoid (Rani et al. 2020), plumbagin (Singh et al. 2020) and astragalosides (Park et al. 2020).

An experimental design is a set of tools for studying a system which includes planning and performing a set of experiments to determine the impact of the studied variables on that system. In such experiments, the obtained valid model is a model that contains valuable information about the effect of experimental conditions on the measurement response rate. The required experiment is performed in such a way in which a large amount of information is obtained from a limited number of experiments. Once the appropriate model is obtained, it can be used to predict future observations within the original design range. Therefore, it is necessary to use an appropriate experimental design to develop and optimize a wide range of laboratory studies. The response surface methodology (RSM), which includes statistical and mathematical tools, was first used in chemical experiments for designing and analyzing response surfaces. The experimental design method and the response surface methodology are closely related together and using the RSM methodology is based on experimental data (Mäkelä 2017). This statistical model is an effective tool for optimizing complex processes and save time during the experimental phase (Menezes Maciel Bindes et al. 2019). In the present study the effect of yeast extract and sampling time on cell growth, azadirachtin, mevalonic acid and squalene accumulation and production were investigated and predicted their effects on azadirachtin, mevalonic acid and squalene accumulation and production by response surface methodology. Also, the effect of yeast extract was studied on the squalene synthase 1 (*SQS1*) and squalene epoxidase 1 (*MOF1*) genes expression. This is the first comprehensive study on gene expression and prediction on the effect of yeast extract on cell suspension culture of neem and so far no study has been done.

## Material and methods

### Plant material and cell suspension culture establishment

The leaves of neem were collected from Bandar Abbas city of Iran. The leaves were surface sterilized with ethanol and sodium hypochlorite and cultured on MS medium containing 1 mg/L picloram and 2 mg/L kinetin. Then cultures were maintained in the growth chamber at 25 ± 2 °C in the dark. The friable calli were transferred to the liquid MS medium with the same concentrations of picloram and kinetin and kept on a rotary shaker at 110 rpm and 26 ± 2 °C in the dark and sub-cultured every 12 days (Farjaminezhad and Garoosi 2019).

### Elicitation with yeast extract

The cell suspension cultures were transferred to 100 mL Erlenmeyer flasks containing 25 mL liquid MS medium supplemented with 1 mg/L picloram and 2 mg/L kinetin with an initial cell density of 2.6 × 10^5^ (SCV = 8%). The stock solution of yeast extract (Merck, Germany) was prepared by dissolving of yeast extract in distilled water and then filtered using a 0.22 µm syringe filter. According to our previous study growth curve (Farjaminezhad and Garoosi 2019), eight days after cell culture different concentrations of yeast extract including 0, 25, 50, 100, 150 and 200 mg/L were added to cell suspension cultures. The cultures were kept on a rotary shaker at 110 rpm and 26 ± 2 °C in the dark and sampling was performed after 2, 4, 6, 8, 10 and 12 days of each treatment.

### Fresh and dry cell weight measurement

Fresh and dry cell weights were measured as described by Godoy-Hernández and Vázquez-Flota (2006) with a little modification. For this purpose the cells were collected by Whatman No. 1 filter paper using Büchner funnel under vacuum condition for 30 s and weighed immediately. Then, the collected cells transferred to the oven at 50 °C for 72 h and weighed immediately for dry cell weight.

### Mevalonic acid, squalene and azadirachtin extraction

Azadirachtin, mevalonic acid, and squalene were extracted by using Rafiq and Dahot (2010) method with some modifications. One milliliter of dichloromethane was added to 100 mg of dried and powdered cells and then sonicated for 25 min at room temperature. The supernatant was collected after centrifugation at 7000 rpm for 15 min. The procedure was repeated for two times. Finally, the dichloromethane was evaporated at 50 °C in a water and the samples were dried as described above. The dried samples then were re-dissolved in 1.50 mL HPLC-grade distilled water and stored at − 20 °C.

### HPLC analysis

The HPLC analysis was performed using a Knauer HPLC–DAD system (DAD detector, Azura, Germany) with a Toso C18 column (TSKgel-ODS, 5 µm, 4.60 × 250 mm, Japan). The mobile phase was acetonitrile:water (10:90) at a flow rate of 0.90 mL/min. The detection wavelength for azadirachtin (Sigma, USA), mevalonic acid (Sigma, USA), and squalene (Sigma, USA) were set at 214, 270 and 195 nm, respectively. The injection volume of samples was 20 μL. The azadirachtin, squalene, and mevalonic acid accumulation were estimated from the standard curve of concentration versus the peak area. Also, the amount of azadirachtin, mevalonic acid, and squalene production was calculated by multiplying the amount of azadirachtin, mevalonic acid, and squalene accumulation in their dry cell weight, respectively (Farjaminezhad and Garoosi 2020).

### RNA extraction, cDNA synthesis and qRT-PCR analysis

For RNA extraction, cDNA synthesis, and qRT-PCR analysis, those samples with the highest amount of azadirachtin accumulation at each concentration of yeast extract were used. Total RNA was extracted using an RNX-Plus kit (Cinaclon, Iran) based on the producer’s protocol. The quantity of extracted RNA was measured by a Nano-Drop 200C spectrophotometer (Thermo Scientific, USA). Then, extracted total RNAs were treated with DNase I, RNase-free (Sinaclon, Iran) according to the producer’s guidance to eliminate remaining genomic DNA. Single-strand cDNA was synthesized by using a mixture of 5 µg of total RNA, 0.50 µg/µL Oligo (dT)_18_ primer (Cinaclon, Iran) and 12.50 μL DEPC-treated water in the tube. The tube was maintained at 65 °C for 5 min and immediately transferred onto ice. Then, 2 μL 10 × reaction buffer (Cinaclon, Iran), 2 μL dNTP Mix 10 mM (Cinaclon, Iran) and 1 µL M-MuLv reverse transcriptase enzyme (200 µ/µL, Cinaclon, Iran) were added into tube and maintained at 42 °C for 60 min. The reaction was terminated by heating the mixture at 70 °C for 10 min. The reverse transcription reaction product was stored at – 20 °C untile qRT-PCR analysis. The qRT-PCR analysis performed by real-time PCR (Applied Biosystems StepOnePlus, USA) with specific primers for squalene synthase (*SQS1*) gene (forward: 5ʹ-GCTGAAAATGGCTGTGAGGC-3ʹ and reverse: GTCAGTCCCGAGCTGTTGAA-3ʹ), squalene epoxidase *1* (*MOF1*) gene (forward: 5ʹ-TCAAATCTGCGCCGTTCTCT-3ʹ and reverse: 5ʹ-AGAATGACATGCCCGTGGTT-3ʹ) and housekeeping 18S ribosomal RNA gene (forward: 5ʹ-CACCACACAACTCTCCCCAT-3ʹ and reverse: 5ʹ-ATCAACCACCGTAGTGTCGC-3ʹ). The qRT-PCR mixture contained 1 µL of synthesized cDNA (50 ng), 7.50 µL SYBR Green Premix Ex Taq II (Takara, Japan), 0.50 µL of 10 µmol of gene-specific primer pairs, and 6 µL of nuclease-free water in a final volume of 15 µL. qRT-PCR conditions consisted of primary denaturation at 95 °C for 2 min, followed by 35 cycles of denaturation at 95 °C for 30 s, annealing and extension steps at 57 °C for 30 s and at 72 °C for 30 s, respectively. Finally, the data were analyzed using a 2^−ΔΔCT^ method (Livak and Schmittgen 2001).

### Statistical analysis and experimental design by response surface methodology (RSM)

The treatment of yeast extract was performed in factorial experiment based on a completely randomized design in triplicate in which the first factor was the different concentrations of yeast extract and the second factor was different sampling times. Data were analyzed by IBM SPSS Statistics software, Version 24.0 (Armonk, NY, USA). The measured indices means compared by using Duncan’s multiple range test at a probability level of 0.01. The qRT-PCR analysis of *SQS1* and *MOF1* genes performed in two biological and two technical replications separately. The mean comparison of relative expression of genes also was carried out using Duncan’s multiple range test at a probability level of 0.01.

Response surface methodology was used to study the effects of independent variables including different concentrations of yeast extract and different sampling time as well as their interaction on accumulation and production of azadirachtin, mevalonic acid, and squalene. The sampling times were selected based on preliminary studies. The central composite design (CCD) with two variables and five different levels (− 2, − 1, 0, + 1, + 2) was used for the optimization of yeast extract concentration and sampling time. A total of 13 experiments were conducted to test the five levels of yeast extract and sampling time with full-factorial CCD. By using coded units, the experimental and predicted values for the azadirachtin, mevalonic acid, and squalene accumulation and production in terms of the different variables of yeast extract and sampling time are presented in Tables 1 and 2. The predicted response was calculated using the quadratic polynomial model. The predicted responses which were calculated by a second-order polynomial (quadratic) model is shown as following:

$$\mathrm{Y}={\upbeta }_{0}+\sum_{\mathrm{i}=1}^{\mathrm{n}}{\upbeta }_{\mathrm{i}}{\mathrm{X}}_{\mathrm{i}}+\sum_{\mathrm{i}=1}^{\mathrm{n}}{\upbeta }_{\mathrm{ii}}{\mathrm{X}}_{\mathrm{i}}^{2}+\sum_{\mathrm{i}=1}^{\mathrm{n}-1}\sum_{\begin{array}{c}j=2\\ j>i\end{array}}{\upbeta }_{\mathrm{ij}}{\mathrm{X}}_{\mathrm{i}}{\mathrm{X}}_{\mathrm{j}},$$

where, Y is the response variable, β_0_ is the average response obtained during replicated experiments of the CCD; β_i_, β_ii_, and β_ij_ are the linear, quadratic, and cross-product effects, respectively; X_i_ and X_j_ are the independent coded variables. Response surface regression coefficient and Analysis of Variance (ANOVA) predicted the effects of independent variables on azadirachtin, mevalonic acid, and squalene accumulation and production from cell suspension culture of neem. The data were analyzed using Design Expert (12.0.0 version) software.

**Table 1:** Experimental and predicted values for azadirachtin and mevalonic acid accumulation and production optimized with central composite design (CCD)

Experiment	Yeast extract (mg/L) A	Sampling time (day) (B)	Azadirachtin accumulation (mg/g DW)		Azadirachtin production (mg/L)		Mevalonic acid accumulation (mg/g DW)		Mevalonic acid production (mg/L)	
			Actual value	Predicted value	Actual value	Predicted value	Actual value	Predicted value	Actual value	Predicted value
1	50 (− 1)	4 (− 1)	6.10	6.30	160.56	147.73	0.12	0.14	3.63	4.02
2	100 (0)	2 (− 2)	**9.89**	**10.10**	**170.37**	**184.63**	0.09	0.07	1.29	1.12
3	50 (− 1)	8 (+ 1)	5.16	5.75	32.44	49.03	0.25	0.24	4.16	4.58
4	0 (− 2)	6 (0)	4.70	4.30	47.88	46.46	0.26	0.26	5.71	**5.50**
5	100 (0)	10 (+ 2)	4.83	4.64	47.22	32.05	0.14	0.15	1.41	1.20
6	100 (0)	6 (0)	6.50	6.46	119.68	121.32	0.28	0.25	5.12	5.26
7	100 (0)	6 (0)	6.05	4.46	108.61	121.32	0.28	0.25	4.97	5.26
8	150 (+ 1)	4 (− 1)	9.59	8.97	144.01	129.24	0.27	0.31	4.19	4.52
9	100 (0)	6 (0)	6.83	6.46	122.59	121.32	0.278	0.25	4.97	5.26
10	200 (+ 2)	6 (0)	4.87	5.28	53.81	54.32	**0.51**	**0.49**	5.62	5.46
11	100 (0)	6 (0)	6.46	6.46	126.96	121.32	0.28	0.25	5.02	5.26
12	100 (0)	6 (0)	6.44	6.46	130.60	121.32	0.18	0.25	**6.97**	5.26
13	150 (+ 1)	8 (+ 1)	4.29	4.06	60.72	75.37	0.28	0.29	3.67	4.04

Bold values indicated the highest amount

**Table 2:** Experimental and predicted values for sequalene accumulation and production optimized with central composite design (CCD)

Experiment	Yeast extract (mg/L) A	Sampling time (day) (B)	Squalene accumulation (mg/g DW)		Squalene production (mg/L)	
			Actual value	Predicted value	Actual value	Predicted value
1	100 (0)	8 (0)	0.01	0.02	0.12	0.22
2	100 (0)	8 (0)	0.01	0.02	0.11	0.22
3	100 (0)	4 (− 2)	0.13	**0.13**	**2.21**	**2.29**
4	100 (0)	12 (+ 2)	0.01	0.01	0.09	0.11
5	100 (0)	8 (0)	0.01	0.02	0.12	0.22
6	50 (− 1)	6 (− 1)	**0.14**	0.11	2.15	1.81
7	200 (+ 2)	8 (0)	0.02	0.01	0.16	0.00
8	150 (+ 1)	6 (− 1)	0.03	0.03	0.2	0.34
9	100 (0)	8 (0)	0.01	0.02	0.11	0.22
10	150 (+ 1)	10 (+ 1)	0.01	0.02	0.09	0.22
11	100 (0)	8 (0)	0.03	0.02	0.41	0.22
12	0 (− 2)	8 (0)	0.06	0.08	0.75	1.01
13	50 (− 1)	10 (+ 1)	0.01	0.00	0.03	0.00

Bold values indicated the highest amount

## Results

### Fresh and dry cell weight

**Fig. 1:**
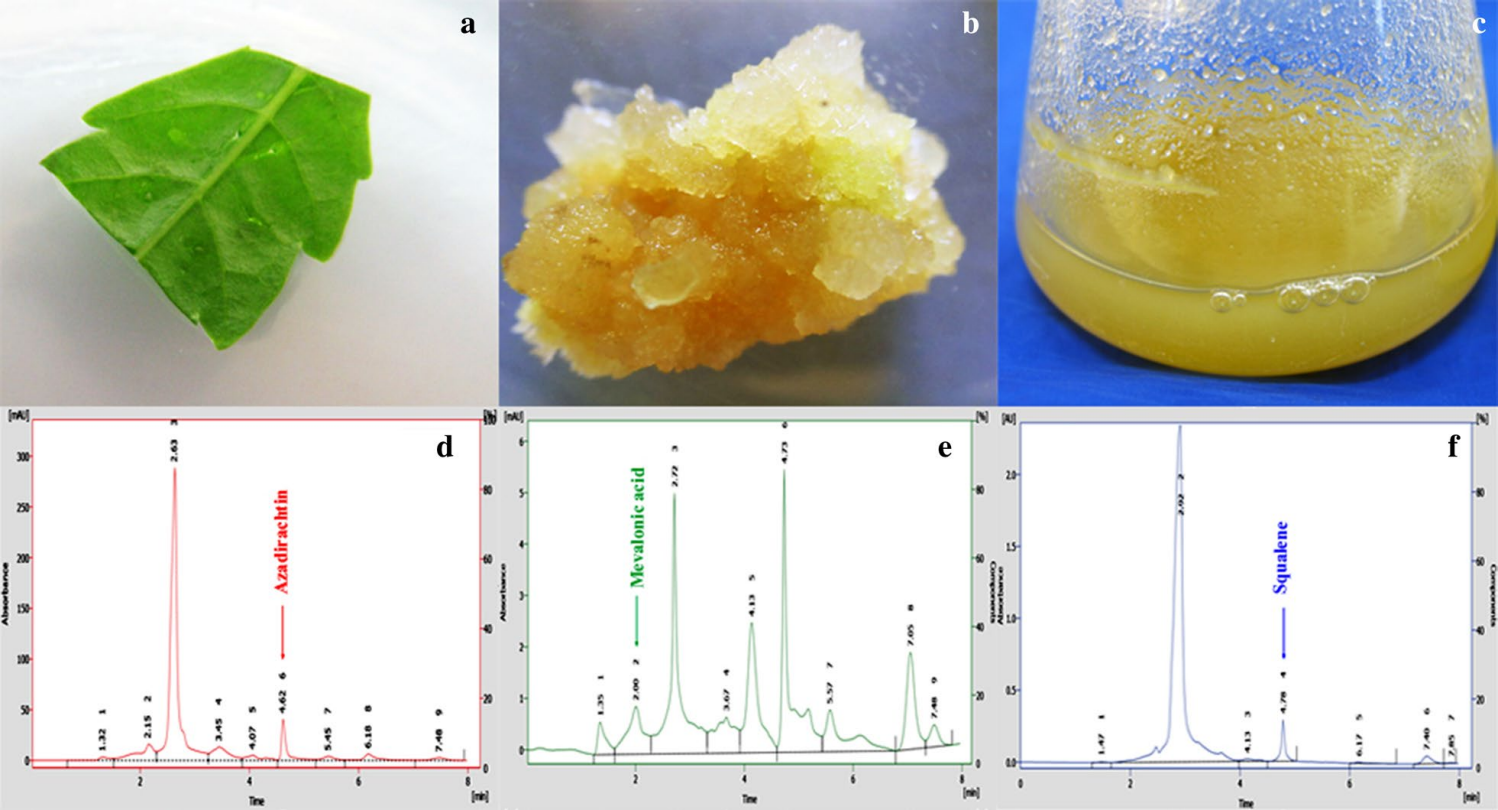
The leaf explant (**a**), callus production (**b**), established cell suspension culture of *A. indica* (**c**), and HPLC chromatograms of azadirachtin (**d**), mevalonic acid (**e**), and squalene (**f**)

Figure 1a–c shows the leaf explant, callus production, and cell suspension culture of *A. indica*. The obtained results demonstrated that different yeast extract concentrations, sampling time, and their interactions affected both the fresh and dry cell weight (Table 3). The investigation of the main effect of the application of yeast extract showed that the fresh and dry cell weight decreases; so that, the use of yeast extract alone and regardless of the sampling time had a negative effect on neem cell suspension culture growth. The most suitable condition for neem cell suspension growth was control without any concentrations of yeast extract. Under these conditions, the mass of fresh and dry cell weight were maximized, which were 413.41 g/L and 14.47 g/L, respectively. Based on results, by addition 25, 50, 100, 150 and 200 mg/L of yeast extract in comparing to the control, the fresh cell weight reduced 29.61, 35.61, 27.73, 45.52 and 48.66% and the dry cell weight reduced 22.53, 25.57, 8.64, 27.99 and 33.86%, respectively. The sampling time of 6 and 4 days gave the highest fresh cell weight and dry cell weight of 410.69 g/L and 16.23 g/L. The fresh cell weight increased from the 2nd day to the 6th day of sampling time and decreased from the 6th day to the 12th day; meanwhile, the dry cell weight increased from the 2nd day to the 4th day of sampling time and decreased from the 4th day to the 12th day. However, the fresh cell weight on the 6th day of sampling was 47.00, 1.66, 88.52, 111.52 and 104.56% higher than those on 2nd, 4th, 8th, 10th and 12th days of sampling and also dry cell weight on 4th day of sampling time was 22.67, 9.90, 80.05, 95.30 and 99.96% higher than those on 2nd, 6th, 8th, 10th and 12th days of sampling time. By interaction study of the effect of different concentrations of yeast extract and sampling times, it was found the highest fresh and dry cell weight were 580.25 g/L and 21.01 g/L on 6th day after addition 100 mg/L of yeast extract and on 4th day after using 50 mg/L of yeast extract. In this treatments, fresh cell weight increased 9.00, 50.86, 23.23, 109.97 and 146.06% compared to control, 25, 75, and 100 mg/L of yeast extract at same sampling time and the dry cell weight increased 24.17, 34.33, 21.37, 40.91 and 81.43% compared to the control, 25, 75, and 100 mg/L of yeast extract at same sampling time (Additional file 1: Table S1).

**Table 3:** Analysis of variance of the effect of yeast extract and sampling times on measured indices in cell suspension culture of neem

S.O.V		Yeast extract (YE)	Sampling time (ST)	YE × ST	Error	CV (%)
df		5	5	25	72	
Mean of square	Fresh cell weight	93,350.82**	179,438.79**	33,712.14**	2256.49	16.70
	Dry cell weight	61.88**	229.58**	24.07**	1.35	9.27
	Azadirachtin accumulation	72.18**	37.009**	11.93**	0.60	11.23
	Azadirachtin production	10,274.50**	27,632.98**	3433.52**	115.54	13.24
	Mevalonic acid accumulation	0.88**	1.47**	0.51**	0.007	24.67
	Mevalonic acid production	177.95**	314.43**	108.41**	1.51	26.89
	Squalene accumulation	0.006**	0.02**	0.006**	0.00003	12.55
	Squalene production	2.30**	6.87**	1.98**	0.02	21.88

* and **significant at 5% and 1% probability level, respectively

### Azaidrachtin accumulation and production

**Fig. 2:**
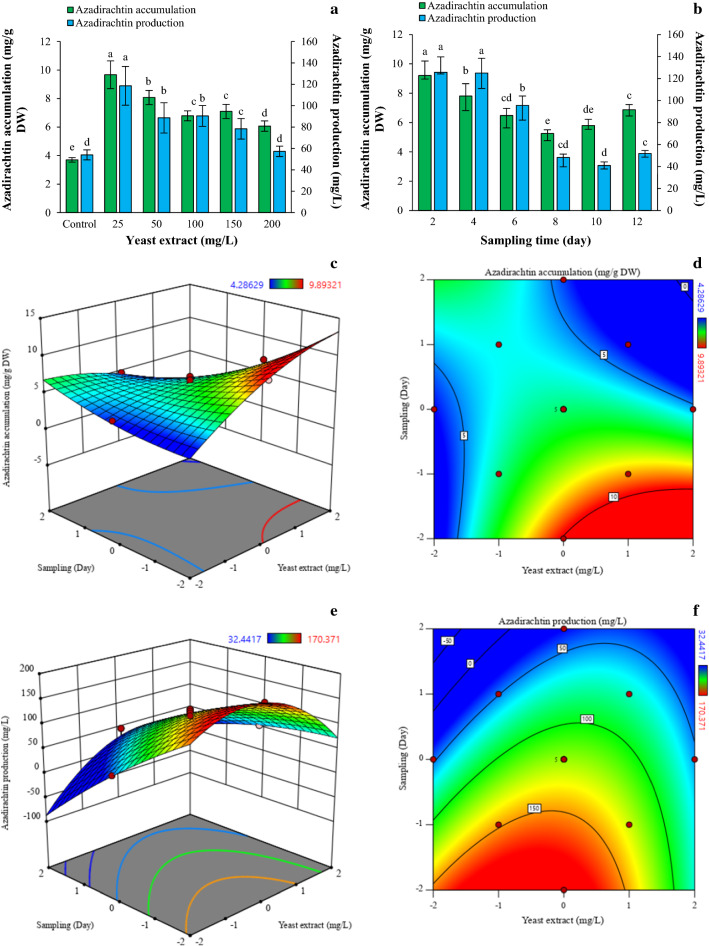
The effect of different concentrations of yeast extract and sampling times (**a**, **b**) and response surface and contour plots for azadirachtin accumulation (**c**, **d**) and azadirachtin production (**e**, **f**)

The HPLC chromatogram of azadirachtin showed in Fig. 1d. The analysis demonstrated that the applied concentrations of yeast extract, sampling times, and their interaction significantly stimulated the azadirachtin accumulation and production in treated cells compared to the control (Table 3). Accumulation and production of azadirachtin showed a dose-dependent response to the yeast extract. A more increase in azadirachtin accumulation and production was observed at 25 mg/L yeast extract. Yeast extract at the lower concentration of 25 mg/L showed the highest azadirachtin accumulation and production which was 9.67 mg/g DW and 118.53 mg/L. The azadirachtin accumulation and production from control treatment to 25 mg/L of yeast extract increased and then decreased along with increasing yeast extract concentration from 25 to 100 mg/L. At the 25 mg/L of yeast extract the azadirachtin accumulation was 161.57, 19.79, 42.45, 36.34 and 59.37% and the azadirachtin production was 119.74, 33.80, 31.16, 51.28 and 106.81% higher than control, 50, 100, 150, and 200 mg/L (Fig. 2a). In terms of sampling time, the highest azadirachtin accumulation and production were 9.20 mg/g DW and 125.65 mg/L observed at 2 days after treatment. On the 2nd day of sampling time, the azadirachtin accumulation increased 17.94, 42.22, 75.44, 58.97 and 34.02% and azadirachtin production increased 0.53, 31.53, 160.65, 207.37 and 142.42% compared to the 2nd, 4th, 8th, 10th, and 12th days, respectively (Fig. 2b). The effect of different concentrations of yeast extract along with different sampling times is shown in Additional file 1: Table S1. The best conditions for induction of azadirachtin accumulation and production were an application of 25 mg/L for 2 days (16.08 mg/g DW) and 4 days (219.78 mg/L), respectively. Under these conditions, the azadirachtin accumulation increased 462.24, 42.43, 66.11, 82.93 and 146.62% compared to the control, 50, 100, 150, and 200 mg/L yeast extract the 2nd day of sampling and the azadirachtin production increased 302.16, 54.09, 96.23, 51.01 and 191.79% compared to the control, 50, 100, 150 and 200 mg/L at same day, respectively (Additional file 1: Table S1).

### Model for predicting of azadirachtin accumulation and production

The sampling times of 2, 4, 6, 8 and 10 days were chosen to RSM analysis and prediction of azadirachtin accumulation and production. The combined effects of different concentrations of yeast extract and sampling times were investigated by the response surface methodology using a central composite design (CCD). The specific interaction of different concentrations of yeast extract and sampling times with the measured and predicted response values of azadirachtin accumulation and production are shown in Table 1. In this study, the experiment No. 2 in application of 100 mg/L of yeast extract and sampling on the 2nd day had the highest amount of azadirachtin accumulation (9.89 mg/g DW) and the experiment No. 13 in using of 150 mg/L of yeast extract and sampling on the 8th day had the lowest azadirachtin accumulation (4.29 mg/g DW). Also, experiment No. 2 with an application of 100 mg/L of yeast extract and sampling time of 2 days had the highest azadirachtin production (170.37 mg/L) and experiment No. 3 with application of 50 mg/L yeast extract and sampling time of 8 days had the lowest azadirachtin production (32.44 mg/L).

Analysis of variance (ANOVA) of the results of the response surface model was presented in Table 4. In the analysis of variance of CCD, the coefficient of determination of the model for azadirachtin accumulation and production were 95.76 and 94.08%, respectively; which indicates that these actual levels can correspond to the predicted levels. Also, the *p*-value of the models were significant and the proposed models were appropriate. Therefore, the following formulas were calculated to predict the azadirachtin accumulation and production using yeast extract and sampling times:

$${\text{Azadirachtin accumulation }}\left( {{\text{mg}}/{\text{g DW}}} \right) \, = { 6}.{46 } + \, 0.{\text{2452 A }}{-}{ 1}.{\text{36 B }}{-}{ 1}.0{\text{9 AB }}{-} \, 0.{\text{4178 A}}^{{2}} + \, 0.{\text{2269 B}}^{{2}}.$$

$${\text{Azadirachtin production }}\left( {{\text{mg}}/{\text{L}}} \right) \, = { 121}.{32 } + { 1}.{\text{97 A }}{-}{ 38}.{\text{14 B }} + { 11}.{\text{21 AB }}{-}{ 17}.{\text{73 A}}^{{2}} {-}{ 3}.{\text{25 B}}^{{2}}.$$

**Table 4:** Analysis of variance of azadirachtin, mevalonic acid and squalene accumulation and production in the central composite design under influence of yeast extract and sampling times

Source	df	Mean of square					
		Azadirachtin accumulation	Azadirachtin production	Mevalonic acid accumulation	Mevalonic acid production	Squalene accumulation	Squalene production
Model	5	6.95^s^	5058.59^s^	0.02^s^	5.46^s^	0.004^s^	1.33^s^
A: Yeast extract	1	0.72^ns^	46.34^ns^	0.04^s^	0.001^ns^	0.003^s^	0.75^s^
B: Sampling time	1	22.31^s^	17,458.30^s^	0.005^ns^	0.005^ns^	0.01^s^	3.55^s^
AB	1	4.76^s^	502.37^ns^	0.003^ns^	0.27^ns^	0.003^s^	0.93^s^
A^2^	1	4.00^s^	7206.08^s^	0.02^s^	0.07^ns^	0.001^ns^	0.12^ns^
B^2^	1	1.18^ns^	241.38^ns^	0.03^s^	24.06^s^	0.004^s^	1.39^s^
Residual	7	0.22	227.39	0.002	0.56	0.0002	0.06
Lack of fit	3	0.41^ns^	436.22^ns^	0.001^ns^	0.28^ns^	0.0003^ns^	0.10^ns^
Pure error	4	0.08	70.77	0.002	0.76	0.0001	0.02
R^2^ (%)		95.76	94.08	91.67	87.53	94.20	94.48
R_2_^adj^ (%)		92.73	89.85	85.71	78.63	90.05	90.55
Adeq precision		18.94	14.89	15.79	8.65	14.49	15.84

^s^ and ^ns^significant and non-significant, respectively

### Optimization of the response surface of azadriachtin accumulation and production

The interaction between different yeast extract concentrations and different sampling times is shown in Fig. 2. Increasing the concentration of yeast extract along with increasing the exposure time to 6 days had a significant effect on the azadirachtin accumulation. The best yeast extract concentrations and sampling times for maximum azadirachtin accumulation were between 100 and 200 mg/L and 2–4 days. The highest amount of azadirachtin accumulation in this analysis was obtained in application of 100 mg/L of yeast extract after 2 days of sampling time. However, the optimal conditions for maximizing the azadirachtin accumulation (13.607 mg/g DW) predicted two days after application of 245 mg/L of yeast extract (Fig. 2c, d). Also, the results indicated that azadirachtin production depends on sampling time. According to Fig. 2e, f, at all concentrations of yeast extract, azadirachtin production gradually decreases with increasing sampling time. The highest azadirachtin production is achieved by applying 0–150 mg/L of yeast extract and sampling time of 2–6 days. In this analysis, the highest azadirachtin production was 2 days after application of 100 mg/L of yeast extract, but it is predicted that the highest azadirachtin production with 71 mg/L is obtained by culturing of cell for 2 days at medium containing 190.50 mg/L of yeast extract.

### Mevalonic acid accumulation and production

**Fig. 3:**
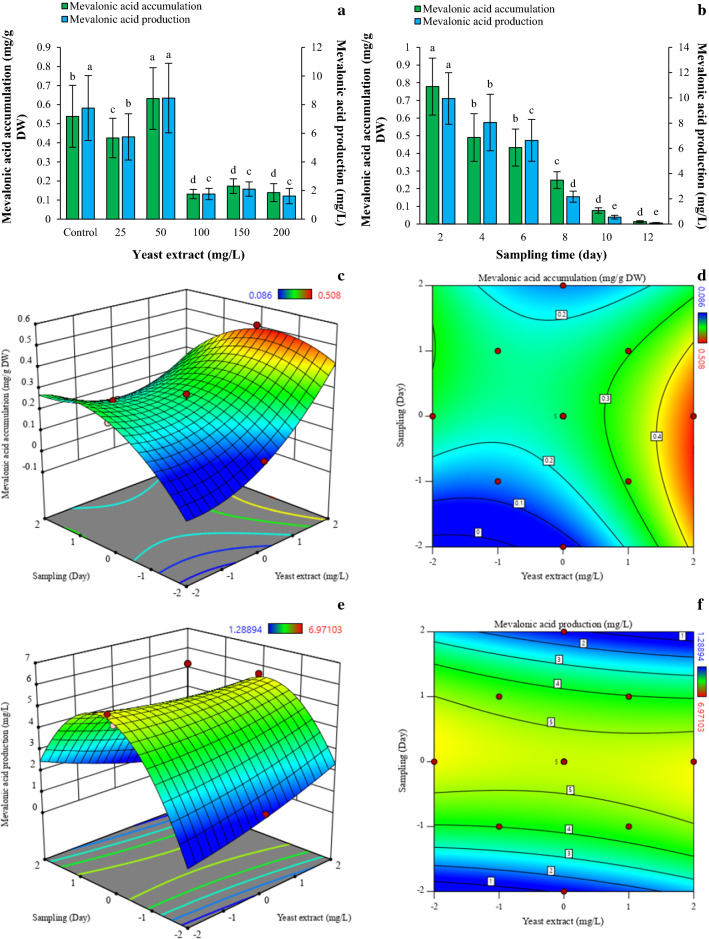
The effect of different concentrations of yeast extract and sampling times (**a**, **b**) and response surface and contour plots for mevalonic acid accumulation (**c**, **d**) and mevalonic acid production (**e**, **f**)

The HPLC chromatogram of mevalonic acid illustrated in Fig. 1e. The mevalonic acid accumulation and production were significantly changed under different yeast extract concentrations, sampling times, and their interactions (Table 3). Among the studied concentrations of yeast extract, 50 mg/L increased the accumulation of mevalonic acid compared to the control; and 25, 100, 150 and 200 mg/L of yeast extract decreased it in comparison to the control. With increasing the concentration of yeast extract in the culture medium from 0 to 25 mg/L, the accumulation of mevalonic acid decreased, and then with increasing the concentration of yeast extract to 50 mg/L it was increased and with increasing the concentration of yeast extract form 50 mg/L to 200 mg/L, its amount decreased again. The highest amount of mevalonic acid accumulation (0.63 mg/g DW) was obtained at 50 mg/L yeast extract, which was 17.31% higher than the control and 48.71, 381.59, 265.15 and 353.86% higher than the 25, 100, 150 and 200 mg/L of yeast extract, respectively. Therefore, higher concentrations of yeast extract in the culture medium prevented the mevalonic acid accumulation. In relation to the production of mevalonic acid, it was observed that with increasing the yeast extract concentration from 0 to 25 mg/L the production of mevalonic acid decreased and with increasing the concentration of yeast extract from 25 to 50 mg/L it was increased; however, the difference between control and application of 50 mg/L yeast extract was not statistically significant. The highest mevalonic acid production (8.45 mg/L) was obtained at 50 mg/L of yeast extract. By adding 50 mg/L of yeast extract to culture medium, the mevalonic acid production increased 8.94, 47.06, 381.84, 302.82 and 424.19% compared to the control and 25, 100, 150 and 200 mg/L of yeast extract, respectively (Fig. 3a). Therefore, the application of moderate concentrations of yeast extract had a positive effect on the accumulation and production of mevalonic acid. By investigating the effect of sampling times, we founded that prolonged exposure of neem cell suspension culture with yeast extract reduces the mevalonic acid accumulation. The highest amount of mevalonic acid accumulation with an average of 0.78 mg/g DW was observed on the second day of sampling. In general, two days after treatment the amount of mevalonic acid accumulation was 55.88, 79.76, 213.19, 915.48 and 5525.94% higher than the 4th, 6th, 8th, 10th, and 12th days, respectively. Also, between different sampling times, the highest mevalonic acid production (9.95 mg/L) was observed on the 2nd day of sampling. By culturing of neem cells for 2 days, the mevalonic acid production was increased 23.63, 50.01, 359.17, 1724.97 and 11,214.56% compared to days 4, 6, 8, 10, and 12, respectively (Fig. 3b). The interactions of yeast extract concentrations and sampling times showed that the highest mevalonic acid accumulation (1.75 mg/g DW) and production (23.77 mg/L) were obtained two days after application of 50 mg/L yeast extract. In this conditions, mevalonic acid accumulation was 8.92, 212.90, 1954.11, 334.27 and 2716.13% higher than the control, 25, 100, 150, and 200 mg/L of yeast extract and mevalonic acid production was 29.82, 216.93, 1801.60, 370.69 and 525.53% higher than the 25, 50, 75, and 100 mg/L of yeast extract on same day (Additional file 1: Table S1).

### Model for predicting of mevalonic acid accumulation and production

Based on the results, the sampling times of 2, 4, 6, 8, and 10 days were selected for RSM analysis and prediction of mevalonic acid accumulation and production. The specific interaction of different concentrations of yeast extract and sampling times with the measured and predicted response values of mevalonic acid accumulation and production is shown in Table 1. Experiment No. 10 (6 days after addition of 200 mg/L of yeast extract) had the highest amount of mevalonic acid accumulation (0.51 mg/g DW) and experiment No. 2 (two days after application of 100 mg/L yeast extract) had the lowest of mevalonic acid accumulation. Also, experiment No. 12 with the application of 100 mg/L of yeast extract for 6 days had the highest amount of mevalonic acid production (6.97 mg/g DW) and experiment No. 2 with using 100 mg/L of yeast extract for two days produced the lowest of mevalonic acid production. Analysis of variance results revealed that the coefficient of determination (R^2^) of the model for mevalonic acid accumulation and production were 91.67 and 87.53%, respectively; which indicates that 91.67 and 87.53% of the actual value can correspond to the predicted value. Also, the *p*-value of the models were significant and the proposed models were appropriate (Table 4). Therefore, the following formulas were calculated to predict the mevalonic acid accumulation and production using yeast extract and sampling times:

$${\text{Mevalonic acid accumulation }}\left( {{\text{mg}}/{\text{g DW}}} \right) \, = \, 0.{2513 } + \, 0.0{\text{569 A }} + \, 0.0{\text{199 B }}{-} \, 0.0{\text{289 AB }} + \, 0.0{\text{313 A}}^{{2}} {-} \, 0.0{\text{364 B}}^{{2}}.$$

$${\text{Mevalonic acid production }}\left( {{\text{mg}}/{\text{L}}} \right) \, = { 5}.{26 }{-} \, 0.0{1}0{\text{1 A }} + \, 0.0{21}0{\text{ B }}{-} \, 0.{\text{2599 AB }} + \, 0.0{\text{543 A}}^{{2}} {-}{ 1}.0{\text{2 B}}^{{2}}.$$

### Optimization of the response surface of mevalonic acid accumulation and production

The interaction between different yeast extract concentrations and different sampling times is shown in Fig. 3. The results showed that yeast extract concentration is the most important factor. Accordingly, gradual increases in the concentration of yeast extract lead to a decrease in the accumulation of mevalonic acid in the neem cell suspension culture. Therefore, in this analysis, the highest amount of accumulation of mevalonic acid was observed at their highest level. Depending on the concentration of yeast extract, sampling time increased or decreased the accumulation of mevalonic acid. According to Fig. 3c, d, the highest amount of mevalonic acid accumulation was obtained 6 days after applying 200 mg/L of yeast extract, but it is predicted that the highest amount of mevalonic acid accumulation with the amount of 0.50 mg/g DW was obtained by culture of the cells for 4.96 days in medium containing 200 mg/L of yeast extract. Also, according to the results of this analysis, the production of mevalonic acid depends on the sampling time. At different concentrations of yeast extract, increasing the duration of cell suspension culture exposure to yeast extract increased the amount of mevalonic production from 2 to 6 days and then decreased. The highest mevalonic acid production was obtained by exposing the cell suspension to 100 mg/L of yeast extract for 6 days. The optimal condition for maximizing the mevalonic acid production is predicted to be a cell suspension culture without yeast extract after 6.54 days, which can produces 5.57 mg/L mevalonic acid.

### Squalene accumulation and production

**Fig. 4:**
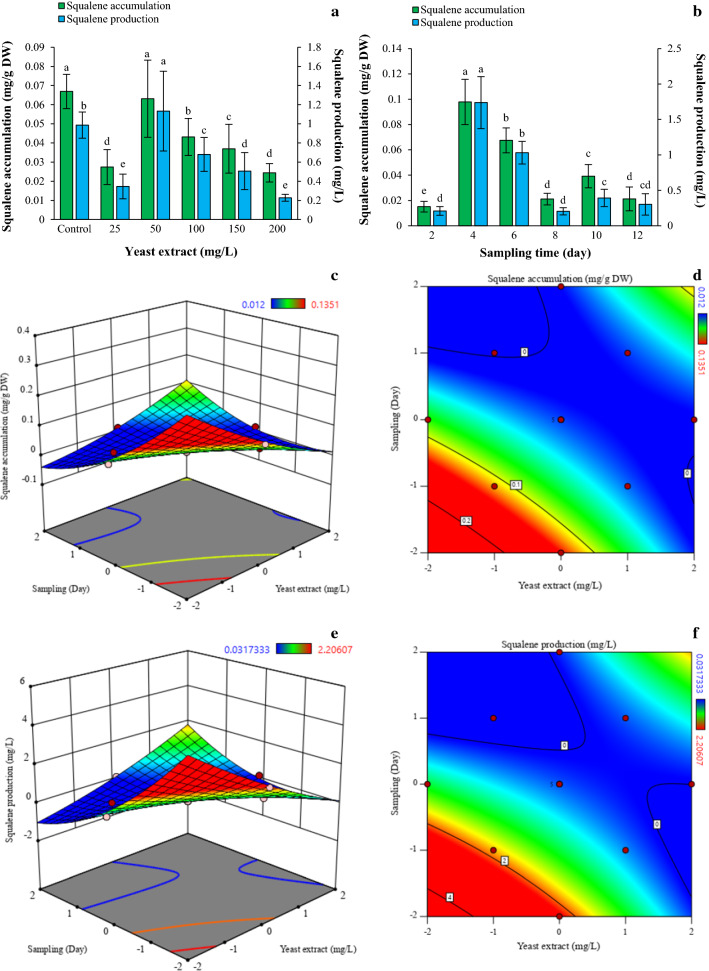
The effect of different concentrations of yeast extract and sampling times (**a**, **b**) and response surface and contour plots for squalene accumulation (**c**, **d**) and squalene production (**e**, **f**)

The HPLC chromatogram of squalene is illustrated in Fig. 1f. The results showed that different concentrations of yeast extract, sampling times, and their interactions had a significant effect on squalene accumulation and production (Table 3). The study of squalene accumulation in cell suspension culture in the presence of yeast extract revealed that yeast extract inhibited squalene accumulation. Increasing the concentration of yeast extract in the culture medium the accumulation of squalene was reduced. With increasing the yeast extract concentration from 25 to 50 mg/L, the squalene accumulation was increased and then decreased. The highest squalene accumulation (0.07 mg/g DW) between different concentrations of yeast extract was obtained at the control condition. With using 25, 50, 100, 150, and 200 mg/L yeast extract squalene accumulation decreased 59.70, 5.97, 35.82, 42.27 and 64.17% than control, respectively. Analysis the data about squalene production revealed that the application of 50 mg/L of yeast extract increased squalene production compared to the control and application of 25, 100, 150, and 200 mg/L decreased it. Among different concentrations of yeast extract, the highest production of squalene (1.13 mg/L) was obtained at 50 mg/L, which was 14.85, 227.71, 66.62, 123.72 and 398.77% higher compared to the control and 25, 100, 150, and 200 mg/L, respectively (Fig. 4a). Between different sampling times, the highest amount of squalene accumulation was 0.10 mg/g DW obtained on the 4th day of sampling time. In general, 4 days after yeast extract treatment the amount of squalene accumulation comparing to 2, 6, 8, 10 and 12 days was 547.87, 44.95, 363.76, 149.67 and 361.09% higher, respectively. Investigation of different sampling times showed that during the first 4 days, the production of squalene in the cell suspension culture of neem was increased and then was decreased on further days. The highest amount of squalene production (1.74 mg/L) was observed on the 4th day of sampling. So that, on the 4th day of sampling time the amount of squalene production in comparing to the 2nd, 6th, 8th, 10th, and 12th days was 732.07, 68.63, 748.53, 343.38 and 474.63% higher, respectively (Fig. 4b). Further study of squalene accumulation and production was performed using the simultaneous examination of different concentrations of yeast extract and sampling times. In this context, the results showed that 4 days after application of 50 mg/L of yeast extract the highest amount of squalene accumulation (0.22 mg/g DW) and production (4.53 mg/L) were obtained, which were 283.93, 1333.3, 69.29, 41.45 and 923.81%; and 377.43, 1817.79, 105.54, 99.47 and 1739.84% higher in comparing to the control, 25, 100, 150, and 200 mg/L, respectively (Additional file 1: Table S1).

### Model for predicting of squalene accumulation and production

The sampling times of 4, 6, 8, 10, and 12 days were selected for RSM analysis and prediction of squalene accumulation and production. The experimental and predicted values of yeast extract-induced squalene accumulation are shown in Table 2. Experiment No. 6 (6 days after application of 50 mg/L yeast extract) had the highest squalene accumulation (0.14 mg/g DW) and experiment No. 10 (150 mg/L of yeast extract after 10 days) had the lowest squalene production (0.01 mg/g DW). Experiment No. 3 with an application of 100 mg/L of yeast extract and sampling time of 4 days had maximum squalene production (2.21 mg/L) and experiment No. 13 with an application of 50 mg/L of yeast extract and sample time of 10 days had the lowest squalene production. The results of the analysis of variance of central composite design showed that the coefficient of determination (R^2^) of the model is 94.20% for squalene accumulation and 94.48% for squalene production. This indicates that 94.20 and 94.48% of the actual value of squalene accumulation and production correspond to the predicted value. Also, the *p*-value of the models were significant for squalene accumulation and production, which indicates the suitability of the models (Table 4). Therefore, mathematical models which were obtained to predict the squalene accumulation and production under yeast extract elicitation were as following:

$${\text{Squalene accumulation }}\left( {{\text{mg}}/{\text{g DW}}} \right) \, = \, 0.0{199 }{-} \, 0.0{\text{169 A }}{-} \, 0.0{3}0{\text{4 B }} + \, 0.0{\text{274 AB }} + \, 0.00{\text{58 A}}^{{2}} + \, 0.0{\text{135 B}}^{{2}}.$$

$${\text{Squalene production }}\left( {{\text{mg}}/{\text{L}}} \right) \, = \, 0.{215}0 \, {-} \, 0.{25}0{\text{9 A }}{-} \, 0.{\text{5438 B }} + \, 0.{\text{4834 AB }} + \, 0.0{\text{725 A}}^{{2}} + \, 0.{\text{2466 B}}^{{2}}.$$

### Optimization of the response surface of squalene accumulation and production

Figure 4 shows the interaction between different yeast extract concentrations and different sampling times. According to the results of the response surface methodology, the squalene accumulation depends on the concentration of yeast extract and the sampling time. The yeast extract concentration is the most important parameter and with increasing that the squalene accumulation has reached a minimum. In the different concentrations of yeast extract except for 200 mg/L, the accumulation of squalene decreased on over times. At 200 mg/L yeast extract, the squalene accumulation was decreased till the tenth day of sampling and then was increased. Therefore, depending on the concentration of yeast extract, sampling time can have a positive or negative effect on squalene accumulation. Based on this analysis results, the highest amount of squalene accumulation was obtained 6 days after application of 50 mg/L yeast extract, but the prediction results showed that the highest squalene accumulation can be obtained at free-yeast extract medium after 4 days, which can accumulates 0.30 mg/g DW of squalene. Also, increasing the yeast extract concentration during low exposure periods reduced squalene production, but increasing yeast extract concentration at high exposure times enhanced squalene production. According to Fig. 4e, f, it can be said that using 0–100 mg/L of yeast extract for 4–8 days can produce an acceptable amount of squalene. It is predicted that the highest amount of squalene production is achieved by long-term exposure to high concentrations of yeast extract.

### qRT-PCR analysis of *SQS1* and *MOF1* genes expression

**Fig. 5:**
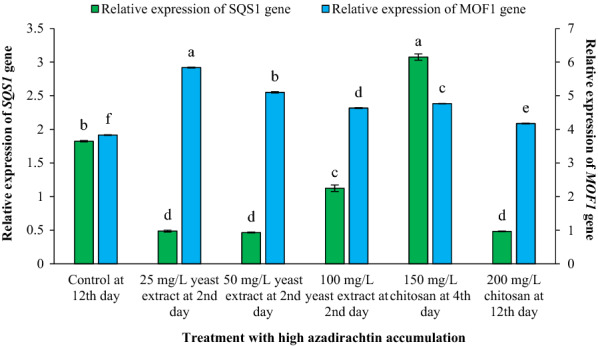
Relative expression profile of *SQS1* and *MOF1* genes normalized with *18S ribosomal RNA* as an internal control

The qRT-PCR analysis presented relative gene expression of *SQS1* gene was significantly increased 68.96% at 4 days application of 150 mg/L of yeast extract compared with the control cells after 12 days. But, after the addition of 25, 50 and 100 mg/L of yeast extract for 2 days, and 200 mg/L of yeast extract for 12 days, the relative expression of *SQS1* gene decreased 73.26, 74.49, 38.29 and 73.53%, respectively compared to the control after 12 days. Also, the application of yeast extract significantly up-regulated *MOF1* gene. The highest relative expression of the *MOF1* gene observed by addition 25 mg/L yeast extract for 2 days, which was 52.41, 14.46, 26.02, 22.51 and 39.90% higher than control on 12 days, 50 mg/L of yeast extract on 2 days, 100 mg/L of yeast extract on 2 days, 150 mg/L of yeast extract on 4 days, and 200 mg/L of yeast extract on 12 days (Fig. 5).

## Discussion

Many researchers have been reported that the use of elicitors is effective in cell growth and increasing bioactive compounds. The age of the cell culture, the duration of exposure to the elicitor, and the type of elicitor have a very effective role in increasing the effect of the elicitors (Açıkgöz 2020; Nazir et al. 2019). Elicitor concentration is an important factor for the elicitation process and the optimum level depends on the plant species (Vasconsuelo and Boland 2007). The studies showed that high concentrations of elicitors trigger the hypersensitive response and leading to cell death (Park et al. 2020). Yeast extract is applied to enhance growth and secondary metabolites production in the cell and hairy root cultures of different plants such as *Artemisia annua* (Putalun et al. 2007) and *Salvia miltiorrhiza* (Yan et al. 2006). In this study, the highest fresh and dry cell weight was obtained 6 days after the addition of 100 mg/L yeast extract. The positive effect of using yeast extract on cell and plant growth and increasing biomass has been reported in some studies. Krstić-Milošević et al. (2017) reported that biotic elicitors such as yeast extract improved root growth and biomass production in *Gentiana dinarica*. Bayraktar et al. (2016) enhanced biomass production of *Stevia rebaudiana* by yeast extract.

The highest azadirachtin accumulation and production were obtained by using 25 mg/L of yeast extract on 2 and 4 days, maximum mevalonic acid accumulation and production observed 2 days after 50 mg/L of yeast extract addition and the highest amount of squalene accumulation and production were achieved 4 days after application of 50 mg/L of yeast extract. The stimulating effect of yeast extract on the isoflavonoid production in *Pueraria candollei* hairy root cultures was reported (Udomsuk et al. 2011). Rani et al. (2020) showed that the utility of yeast extract enhances daidzein and genistein production in the cell culture of *Pueraria candollei*. Sharma and Agrawal (2018) produced 544.60 µg/g DW plumbagin in root cultures of *plumbago zeylanica* L. by using 100 mg/L of yeast extract. Elicitation of *Helianthus tuberosus* L. with 0.25 mg/L of yeast extract increased inulin content 1.18-fold compared to control (Ma et al. 2017). Park et al. (2016) observed the highest amount of rosmarinic acid (4.98 mg/g) by using 750 mg/L of yeast extract in *Agastache rugosa* cell culture. Szopa et al. (2018) increased lignan production in micro-shoot cultures of *Schisandra chinensis* by elicitation with 5000 mg/L of yeast extract on the first day of the growth period and with 1000 and 3000 mg/L on the 20th day.

Elicitors activate the plant’s defense response, causing consecutive cellular and molecular events and activating biosynthetic genes involved in the production of secondary metabolites (Jiao et al. 2016). Various studies have shown the upregulated accumulation of different secondary metabolites by different elicitors such as yeast extract (van der Heijden et al. 1989; Yoon et al. 2000). For example, in the hairy root cultures of *Artemisia annua* L. application of fungal elicitors enhanced artemisinin production and upregulate expression profile of mevalonate and methylerythritol phosphate biosynthetic genes (Ahlawat et al. 2014). These results are consistent with our findings. In this study, the qRT-PCR analysis showed the maximum relative gene expression of *SQS1* and *MOF1* obtained by application of 150 mg/L of yeast extract for 4 days and 25 mg/L of yeast extract for 2 days, respectively. In cell cultures of *Uncaria tomentosa* and *Tobernamontana divaricate*, the activities of enzymes involved in triterpenoid biosynthesis such as IDI and SS are stimulated by elicitors (Flores-Sánchez et al. 2002; Fulton et al. 1994). Ge and Wu (2005) reported that yeast elicitor stimulated HMGR activity in *Salvia miltiorrhiza* hairy root cultures. In cell culture of *A. rugosa* the transcript levels of *HPPR* under yeast extract treatment were higher than the control (Park et al. 2016).

In conclusion, this is the first study on the optimization of yeast extract and sampling time for the production of azadirachtin, mevalonic acid, and squalene in the cell suspension cultures and investigation of *SQS1* and *MOF1* genes expression of neem. Central-composite design (CCD) of Response Surface Methodology (RSM) optimized the effects of yeast extract and sampling time for maximum accumulation and production of azadirachtin, mevalonic acid, and squalene. These results showed that advancement in tissue culture techniques and prediction methods could be applied for the production and enhancement of secondary metabolites. Also, neem cell suspension culture is a suitable strategy for the commercial production of secondary metabolites in in vitro conditions. In addition, in the future, methods for predicting optimal conditions for maximum production of secondary metabolites can reduce the production times and costs of new drug compounds.

## Supplementary Information

**Additional file 1**: **Table S1** Effect of different concentrations of yeast extract and sampling times on measured indices of cell suspension culture of neem.

## Data Availability

All data generated or analysed during this study are included in this published article and its supplementary information files.
