# Supplementary material for: Improvement and prediction of secondary metabolites production under yeast extract elicitation of Azadirachta indica cell suspension culture using response surface methodology
Source: AMB Express. 2021 Mar 17;11:43. doi: 10.1186/s13568-021-01203-x (PMC7967109; doi:10.1186/s13568-021-01203-x)
Supplement: Supplementary file 1 — Additional file 1: Table S1 Effect of different concentrations of yeast extract and sampling times on measured indices of cell suspension culture of neem. [file 13568_2021_1203_MOESM1_ESM.pdf]

AMB Express

Improvement and prediction of secondary metabolites production under yeast extract elicitation of *Azadirachta indica* cell suspension culture using response surface methodology

Reza Farjaminezhad[1] and Ghasem-ali Garoosi[1*]

[1]Department of Biotechnology, Faculty of Agriculture and Natural Resources, Imam Khomeini International University (IKIU), P.O. Box 288, 34149-16818, Qazvin, Islamic Republic of Iran.

[*] Corresponding author e-mail:

E-mail: garoosi@eng.ikiu.ac.ir

Table S1 Effect of different concentrations of yeast extract and sampling times on measured indices of cell suspension culture of neem.

Yeast extract (mg/L)	Sampling time (day)	Fresh cell weight (mg/L)	Dry cell weight (mg/L)	Azadirachtin accumulation (mg/g DW)	Azadirachtin production (mg/L)	Mevalonic acid accumulation (mg/g DW)	Mevalonic acid production (m/L)	Squalene accumulation (mg/g DW)	Squalene production (mg/L)
Control	2	260.53 ± 21.64 $^{g-j}$	11.43 ± 0.15 $^{j-m}$	2.87 ± 0.30 ^{m}	32.66 ± 3.04 ^{mno}	1.60 ± 0.05 ^{b}	18.31 ± 0.33 ^{c}	0.004 ± 0.002 ^{op}	0.05 ± 0.03 ^{j}
	4	429.40 ± 58.94 ^{cd}	16.92 ± 1.48 ^{cde}	3.24 ± 0.06 ^{lm}	54.65 ± 3.79 $^{g-l}$	1.33 ± 0.03 ^{c}	22.50 ± 2.40 ^{ab}	0.056 ± 0.007 ^{e}	0.95 ± 0.12 ^{de}
	6	532.32 ± 67.07 ^{ab}	19.28 ± 2.54 ^{ab}	4.04 ± 0.46 $^{j-m}$	79.21 ± 16.15 ^{de}	0.31 ± 0.03 ^{fg}	5.75 ± 0.26 ^{de}	0.058 ± 0.002 ^{e}	1.13 ± 0.17 ^{d}
	8	312.27 ± 8.66 ^{fgh}	10.46 ± 0.81 ^{lmn}	3.76 ± 0.24 ^{klm}	38.89 ± 0.56 $^{j-o}$	0.00 ± 0.00 ^{l}	0.00 ± 0.00 ^{j}	0.062 ± 0.001 ^{e}	0.65 ± 0.06 ^{fg}
	10	405.21 ± 98.49 ^{cde}	12.98 ± 2.05 ^{hij}	3.85 ± 0.39 ^{klm}	48.41 ± 2.65 $^{h-n}$	0.00 ± 0.00 ^{l}	0.00 ± 0.00 ^{j}	0.114 ± 0.006 ^{d}	1.46 ± 0.16 ^{c}
	12	540.73 ± 8.36 ^{ab}	15.73 ± 0.85 $^{d-g}$	4.44 ± 0.02 ^{jkl}	69.82 ± 4.02 $^{d-g}$	0.00 ± 0.00 ^{l}	0.00 ± 0.00 ^{j}	0.107 ± 0.002 ^{d}	1.69 ± 0.11 ^{c}
25	2	328.19 ± 21.27 ^{efg}	13.36 ± 0.22 ^{hij}	16.08 ± 0.14 ^{a}	214.92 ± 5.25 ^{a}	0.56 ± 0.24 ^{d}	7.50 ± 3.23 ^{d}	0.000 ± 0.000 ^{p}	0.00 ± 0.00 ^{j}
	4	436.94 ± 21.82 ^{cd}	15.64 ± 0.31 $^{d-g}$	14.06 ± 1.07 ^{b}	219.78 ± 17.37 ^{a}	1.20 ± 0.00 ^{c}	18.82 ± 0.41 ^{c}	0.015 ± 0.000 $^{k-n}$	0.24 ± 0.00 ^{hij}
	6	384.63 ± 22.41 ^{def}	13.82 ± 0.46 ^{fgh}	8.53 ± 0.14 ^{de}	117.82 ± 3.53 ^{c}	0.17 ± 0.00 $^{g-k}$	2.37 ± 0.08 $^{g-j}$	0.109 ± 0.000 ^{d}	1.51 ± 0.05 ^{c}
	8	266.99 ± 20.88 $^{g-j}$	10.05 ± 0.21 $^{l-o}$	6.35 ± 0.09 $^{f-j}$	63.78 ± 1.15 $^{e-h}$	0.51 ± 0.00 ^{jkl}	5.15 ± 0.11 ^{ef}	0.021 ± 0.001 ^{ijk}	0.21 ± 0.01 ^{hij}
	10	99.75 ± 2.88 ^{n}	6.17 ± 0.21 ^{qr}	5.88 ± 0.12 ^{ghi}	36.28 ± 1.72 $^{k-o}$	0.11 ± 0.00 ^{c}	0.65 ± 0.02 ^{ij}	0.019 ± 0.000 $^{i-l}$	0.12 ± 0.00 ^{ij}
	12	229.57 ± 2.88 ^{hij}	8.22 ± 0.23 $^{n-q}$	7.13 ± 0.03 ^{fg}	58.61 ± 1.52 $^{f-j}$	0.00 ± 0.00 ^{l}	0.00 ± 0.00 ^{j}	0.000 ± 0.000 ^{p}	0.00 ± 0.00 ^{j}
50	2	250.27 ± 8.56 $^{g-j}$	13.62 ± 0.34 ^{ghi}	11.29 ± 0.04 ^{c}	153.69 ± 3.36 ^{b}	1.75 ± 0.00 ^{a}	23.78 ± 0.59 ^{a}	0.000 ± 0.000 ^{p}	0.00 ± 0.00 ^{j}
	4	480.69 ± 12.16 ^{bc}	21.01 ± 0.36 ^{a}	6.80 ± 0.53 ^{fgh}	142.63 ± 8.99 ^{b}	0.14 ± 0.02 $^{h-l}$	2.90 ± 0.37 $^{f-i}$	0.215 ± 0.016 ^{a}	4.53 ± 0.39 ^{a}
	6	470.88 ± 30.56 ^{bcd}	15.87 ± 0.03 ^{def}	8.94 ± 0.17 ^{de}	141.80 ± 2.94 ^{b}	1.34 ± 0.00 ^{c}	21.20 ± 0.04 ^{b}	0.135 ± 0.000 ^{c}	2.14 ± 0.00 ^{b}
	8	55.71 ± 2.98 ^{n}	5.81 ± 0.43 ^{r}	5.03 ± 0.07 ^{ijk}	29.31 ± 2.43 ^{no}	0.38 ± 0.13 ^{ef}	2.29 ± 0.94 $^{g-j}$	0.009 ± 0.000 $^{l-p}$	0.05 ± 0.00 ^{j}
	10	198.96 ± 3.99 $^{j-m}$	2.34 ± 0.17 ^{s}	9.34 ± 0.01 ^{d}	21.84 ± 1.60 ^{o}	0.17 ± 0.00 $^{g-k}$	0.39 ± 0.03 ^{j}	0.012 ± 0.000 $^{k-o}$	0.03 ± 0.00 ^{j}
	12	140.60 ± 9.08 $^{k-n}$	6.00 ± 0.37 ^{r}	7.03 ± 0.22 ^{fg}	42.24 ± 3.42 $^{i-o}$	0.03 ± 0.00 ^{kl}	0.18 ± 0.01 ^{j}	0.007 ± 0.000 $^{m-p}$	0.04 ± 0.00 ^{j}
100	2	298.53 ± 35.45 ^{ghi}	14.72 ± 0.26 ^{fgh}	9.68 ± 0.15 ^{d}	152.42 ± 9.40 ^{b}	0.09 ± 0.00 ^{kl}	1.26 ± 0.03 ^{hij}	0.047 ± 0.000 ^{f}	0.69 ± 0.01 ^{f}
	4	429.48 ± 5.34 ^{cd}	17.31 ± 0.08 ^{bcd}	6.47 ± 0.25 $^{f-i}$	112.00 ± 4.77 ^{c}	0.00 ± 0.00 ^{l}	0.00 ± 0.00 ^{j}	0.127 ± 0.000 ^{c}	2.20 ± 0.01 ^{b}
	6	580.25 ± 39.16 ^{a}	18.10 ± 0.16 ^{bc}	6.46 ± 0.23 $^{f-i}$	116.96 ± 4.26 ^{c}	0.28 ± 0.00 $^{f-i}$	5.02 ± 0.05 ^{ef}	0.041 ± 0.001 ^{fg}	0.74 ± 0.01 ^{ef}
	8	133.69 ± 7.62 ^{lmn}	8.73 ± 0.28 $^{m-p}$	6.11 ± 0.17 ^{ghi}	53.44 ± 3.09 $^{g-m}$	0.24 ± 0.00 $^{f-j}$	2.13 ± 0.06 $^{g-j}$	0.013 ± 0.000 $^{k-o}$	0.11 ± 0.00 ^{ij}
	10	267.19 ± 13.23 $^{g-j}$	13.92 ± 0.08 ^{fgh}	5.17 ± 0.17 ^{ijk}	62.73 ± 8.04 $^{e-i}$	0.13 ± 0.01 $^{i-l}$	1.77 ± 0.18 $^{g-j}$	0.017 ± 0.000 $^{j-m}$	0.24 ± 0.00 ^{hij}
	12	83.33 ± 2.25 ^{n}	6.56 ± 0.27 ^{qr}	6.84 ± 0.41 ^{fgh}	44.66 ± 1.15 $^{h-n}$	0.05 ± 0.00 ^{kl}	0.35 ± 0.01 ^{j}	0.013 ± 0.000 $^{k-o}$	0.09 ± 0.00 ^{j}
150	2	286.90 ± 10.64 $^{g-j}$	12.83 ± 0.39 $^{h-k}$	8.80 ± 0.18 ^{de}	112.79 ± 2.53 ^{c}	0.39 ± 0.00 ^{ef}	5.05 ± 0.16 ^{ef}	0.028 ± 0.000 ^{hi}	0.36 ± 0.01 ^{hi}
	4	422.16 ± 8.64 ^{cd}	14.91 ± 0.20 $^{e-h}$	9.77 ± 0.09 ^{d}	145.54 ± 0.77 ^{b}	0.27 ± 0.00 $^{f-i}$	4.07 ± 0.07 ^{efg}	0.152 ± 0.000 ^{b}	2.27 ± 0.03 ^{b}
	6	276.34 ± 14.27 $^{g-j}$	10.80 ± 0.25 $^{k-n}$	6.50 ± 1.21 $^{f-i}$	70.35 ± 13.38 $^{e-g}$	0.00 ± 0.00 ^{l}	0.00 ± 0.00 ^{j}	0.025 ± 0.000 ^{ij}	0.27 ± 0.01 ^{hij}
	8	210.36 ± 30.46 $^{i-l}$	9.67 ± 0.27 $^{l-o}$	4.20 ± 0.05 $^{j-m}$	47.23 ± 6.86 $^{h-n}$	0.31 ± 0.03 ^{fg}	3.01 ± 0.34 ^{fgh}	0.005 ± 0.000 ^{nop}	0.05 ± 0.00 ^{j}
	10	73.72 ± 2.54 ^{n}	7.52 ± 0.16 $^{o-r}$	5.39 ± 0.21 ^{hij}	40.58 ± 2.09 $^{j-o}$	0.06 ± 0.00 $^{k-l}$	0.47 ± 0.01 ^{j}	0.011 ± 0.001 $^{k-o}$	0.08 ± 0.00 ^{j}
	12	81.95 ± 2.48 ^{n}	6.79 ± 0.04 ^{por}	7.91 ± 0.30 ^{ef}	53.64 ± 1.82 $^{g-m}$	0.00 ± 0.00 ^{l}	0.00 ± 0.00 ^{j}	0.000 ± 0.000 ^{p}	0.00 ± 0.00 ^{j}
200	2	251.82 ± 27.95 $^{g-j}$	13.43 ± 0.25 ^{hij}	6.52 ± 0.19 $^{f-i}$	87.44 ± 1.04 ^{d}	0.28 ± 0.00 ^{fgh}	3.80 ± 0.07 ^{efg}	0.011 ± 0.000 $^{k-o}$	0.15 ± 0.00 ^{ij}
	4	225.30 ± 8.91 $^{h-k}$	11.58 ± 0.06 $^{i-l}$	6.50 ± 0.70 $^{f-i}$	75.32 ± 8.43 ^{def}	0.00 ± 0.00 ^{l}	0.00 ± 0.00 ^{j}	0.021 ± 0.000 ^{ijk}	0.25 ± 0.00 ^{hij}
	6	219.74 ± 15.48 ^{ijk}	10.74 ± 0.20 $^{k-n}$	4.38 ± 0.25 ^{jkl}	47.08 ± 3.40 $^{h-n}$	0.51 ± 0.00 ^{de}	5.45 ± 0.11 ^{de}	0.036 ± 0.000 ^{gh}	0.39 ± 0.01 ^{hi}
	8	328.07 ± 9.51 ^{efg}	9.36 ± 0.13 $^{g-j}$	6.03 ± 0.51 ^{ghi}	56.59 ± 5.40 $^{f-k}$	0.05 ± 0.00 ^{kl}	0.42 ± 0.01 ^{j}	0.016 ± 0.000 $^{j-m}$	0.15 ± 0.00 ^{ij}
	10	120.14 ± 0.57 ^{mn}	6.92 ± 0.12 ^{pqr}	5.12 ± 0.23 ^{ijk}	35.45 ± 1.88 $^{l-o}$	0.00 ± 0.00 ^{l}	0.00 ± 0.00 ^{j}	0.062 ± 0.001 ^{e}	0.43 ± 0.01 ^{gh}
	12	128.43 ± 8.15 ^{lmn}	5.41 ± 0.22 ^{r}	7.86 ± 1.48 ^{ef}	42.00 ± 6.89 $^{i-o}$	0.00 ± 0.00 ^{l}	0.00 ± 0.00 ^{j}	0.000 ± 0.000 ^{p}	0.00 ± 0.00 ^{j}
